# The efficacy and safety of thalidomide in the treatment of refractory Crohn's disease in adults: a double-center, double-blind, randomized–controlled trial

**DOI:** 10.1093/gastro/goac052

**Published:** 2022-10-20

**Authors:** Xiang Peng, Zi-Wen Lin, Min Zhang, Jia-Yin Yao, Jun-Zhang Zhao, Pin-Jin Hu, Qian Cao, Min Zhi

**Affiliations:** Department of Gastroenterology, The Sixth Affiliated Hospital, Sun Yat-sen University, Guangzhou, Guangdong, P. R. China; Guangdong Provincial Key Laboratory of Colorectal and Pelvic Floor Diseases, The Sixth Affiliated Hospital, Sun Yat-sen University, Guangzhou, Guangdong, P. R. China; Department of Gastroenterology, Sir Run Run Shaw Hospital Affiliated to Zhejiang University, Hangzhou, Zhejiang, P. R. China; Department of Gastroenterology, The Sixth Affiliated Hospital, Sun Yat-sen University, Guangzhou, Guangdong, P. R. China; Guangdong Provincial Key Laboratory of Colorectal and Pelvic Floor Diseases, The Sixth Affiliated Hospital, Sun Yat-sen University, Guangzhou, Guangdong, P. R. China; Department of Gastroenterology, The Sixth Affiliated Hospital, Sun Yat-sen University, Guangzhou, Guangdong, P. R. China; Guangdong Provincial Key Laboratory of Colorectal and Pelvic Floor Diseases, The Sixth Affiliated Hospital, Sun Yat-sen University, Guangzhou, Guangdong, P. R. China; Department of Gastroenterology, The Sixth Affiliated Hospital, Sun Yat-sen University, Guangzhou, Guangdong, P. R. China; Guangdong Provincial Key Laboratory of Colorectal and Pelvic Floor Diseases, The Sixth Affiliated Hospital, Sun Yat-sen University, Guangzhou, Guangdong, P. R. China; Department of Gastroenterology, The Sixth Affiliated Hospital, Sun Yat-sen University, Guangzhou, Guangdong, P. R. China; Guangdong Provincial Key Laboratory of Colorectal and Pelvic Floor Diseases, The Sixth Affiliated Hospital, Sun Yat-sen University, Guangzhou, Guangdong, P. R. China; Department of Gastroenterology, Sir Run Run Shaw Hospital Affiliated to Zhejiang University, Hangzhou, Zhejiang, P. R. China; Department of Gastroenterology, The Sixth Affiliated Hospital, Sun Yat-sen University, Guangzhou, Guangdong, P. R. China; Guangdong Provincial Key Laboratory of Colorectal and Pelvic Floor Diseases, The Sixth Affiliated Hospital, Sun Yat-sen University, Guangzhou, Guangdong, P. R. China

**Keywords:** Crohn’s disease, thalidomide, refractory, clinical remission

## Abstract

**Background:**

Thalidomide is applied in therapy for refractory Crohn's disease (CD) in adults, but systematic and rigorous clinical evidence is scant. The aim was to provide theoretical references for the efficacy of thalidomide in the therapy for refractory CD in adults.

**Methods:**

A double-center, double-blind, placebo-controlled, randomized clinical trial of refractory CD in adults in two inflammatory bowel disease centers in China. In the double-blind trial, patients were randomly assigned to 100 mg of thalidomide or placebo daily for 8 weeks. The primary outcome was considered as the clinical remission rate calculated based on the Crohn's disease activity index at the eighth week following thalidomide or placebo treatment. In open label, non-response to placebo was additionally treated with 8 weeks of thalidomide; all responders were continuously treated with thalidomide until the 48th week.

**Results:**

Twenty-five patients were randomly assigned to each group. At the eighth week, the clinical remission rate in the thalidomide group was significantly higher than that in the placebo group (68.0% [17/25] vs 16.0% [4/25]; relative risk, 4.2; 95% confidence interval, 1.8–10.9, *P *<* *0.001). After a 48-week follow-up, the continuous treatment rate of thalidomide was 46.3% (19/41). Adverse events during the whole process were reported in 58.5% of patients, mainly involving drowsiness, rash, and peripheral neuropathy that were mild and tolerable.

**Conclusion:**

Thalidomide can be used in the induction and maintenance therapy of refractory CD in adults. And it could be one of the treatment options for refractory CD.

## Introduction

Crohn's disease (CD) is an incurable chronic non-specific inflammatory bowel disease associated with environmental, genetic, infectious, and immune factors. The incidence and prevalence of CD have increased sharply throughout the world, especially in Asia [1]. Current therapeutic strategies for CD are always designed to induce and maintain clinical remission and prevent complications. Various biological agents, such as antitumor necrosis factor-α monoclonal antibodies, have emerged to enhance the response of CD patients and alleviate relevant symptoms [2]. Nevertheless, ∼25% of CD patients are primary non-responders to infliximab [3]. Annually, ∼13% of CD patients turn into secondary non-responders [4]. The annual rate of secondary adalimumab failure even reaches 20.3% [5]. This failure may involve mechanisms of drug action and immune-mediated and non-immune-mediated pharmacokinetics [6]. Moreover, most biological agents are delivered through intravenously or subcutaneously rather than using the intestinal route, which is much more inconvenient [7]. Long-term use of biological agents also increases patients’ susceptibility to secondary malignant tumors [8]. The high price of biological agents significantly limits their extensive application. Therefore, refractory CD remains a great medical challenge, especially in developing countries.

In the 1950s, thalidomide was once used as a sedative and antiemetic drug in the treatment of morning sickness but was later removed from the market because of teratogenic adverse reactions. In 1965, an Israeli dermatologist successfully cured skin lesions of leprosy patients using thalidomide, suggesting its potential anti-inflammatory effect [9]. Thalidomide was approved by the Food and Drug Administration (FDA) in 1998 as an immunomodulator for erythema nodosum leprosum [8, 10], heralding a series of clinical trials thereafter. It is reported that thalidomide impairs the stability of tumor necrosis factor-α (TNF-α) mRNA, thus accelerating its degradation [11]. Moreover, thalidomide induces the production of T helper 2 (Th2) and inhibits the production of T helper 1 (Th1) [7, 11]. Through suppressing the activity of nuclear factor-kappaB (NF-κB), thalidomide blocks the translation of interleukin-12 (IL-12); reduces relative levels of IL-2, interferon-γ, and integrin; and triggers the production of IL-4 and IL-5, thereafter attenuating the migratory capacity of white blood cells, inhibiting the phagocytosis of multinucleated cells and alleviating vascular damage [11]. Thalidomide also downregulates intercellular adhesion molecule-1 to inhibit the regeneration of small intestinal microvascular endothelial cells [12]. It is therefore speculated that thalidomide may be effective for CD patients who cannot benefit from biological agents. A previous study has shown that thalidomide alleviates symptoms and signs of children and adolescents with CD [13]. To the best of our knowledge, the therapeutic efficacy of thalidomide in CD adults has only been reported in open-label trials [14, 15] and retrospective studies [11, 16, 17]. For the first time, we performed this double-center, double-blind, randomized, placebo-controlled study to assess the efficacy and safety of thalidomide in inducing the remission of refractory CD in adults.

## Methods

### Study design and patient recruitment

This was a double-center, double-blinded, placebo-controlled, random clinical trial and the allocation ratio was 1:1. Patients paying consecutive visits for refractory and active CD at the Sixth Affiliated Hospital, Sun Yat-sen University (Guangzhou, China) and Sir Run Run Shaw Hospital, Zhejiang University School of Medicine (Hangzhou, China) from August 2016 to January 2020 were recruited. All recruited patients needed to meet all the inclusion criteria and none of the exclusion criteria.

Inclusion criteria: (i) diagnosis of CD according to the guidelines proposed by the European Crohn’s and Colitis Organization in 2015; (ii) aged 18–50 years; (iii) have refractory CD; (iv) colonoscopy-revealed terminal ileum or colorectal lesion; (v) active stage of CD; (vi) informed consent for clinical trial of thalidomide therapy. Refractory CD was defined as treatment failure and/or intolerance to steroids, immunosuppressants, or biological agents. CD in the active stage was defined as a minimum Crohn's disease activity index (CDAI) of 150 points. Treatment failure was defined as meeting any one of the following criteria: (i) CD still in the active stage after a 4-week (or longer) oral administration of ≥0.75 mg/kg/d prednisone or the dose of prednisone could not be reduced to 10 mg/d after a 3-month treatment although CD was relieved; (ii) active CD maintained after a 4-month (or longer) oral administration of thiopurine drugs (azathioprine ≥2 mg/kg/d or 6-mercaptopurine ≥1 mg/kg/d) combined with or without steroids or non-response to the maintenance therapy; (iii) active CD maintained after a 3-month (or longer) intramuscular or subcutaneous injection of methotrexate ≥25 mg/week combined with or without steroids or non-response to the maintenance therapy; (iv) active CD maintained after induction therapy with infliximab for 14 weeks or non-response to the maintenance therapy; (v) active CD maintained after a 12-week induction therapy with adalimumab six times or non-response to the maintenance therapy.

Exclusion criteria: (i) patients showed symptoms of digestive tract obstruction; (ii) women were pregnant or lactating or planning for pregnancy during the study; (iii) patients with central nervous system diseases; (iv) patients showed cardiopulmonary, liver, or kidney insufficiency; (v) patients showed malignant tumors; (vi) patients showed active tuberculosis, active viral hepatitis, Epstein-Barr virus infection, severe immunodeficiency, organ transplantation history, or severe infection; (vii) the last infliximab treatment was performed within the previous 8 weeks; (viii) the last adalimumab treatment was performed within the previous 4 weeks; (ix) patients took steroids for >20 mg/d; (x) patients participated in other clinical trials.

### Interventions

The patients enrolled were then randomly assigned into thalidomide group and placebo group for 8 weeks. Based on the results of previous retrospective studies, patients were recommended to take 100 mg/d thalidomide (or placebo). After an 8-week treatment of thalidomide or placebo, those in the thalidomide group who did achieve clinical response were continuously treated with 100 mg/d thalidomide for maintenance therapy until the 48th week in open label. The patients in the placebo group who did not achieve clinical remission were switching to thalidomide treatment (100 mg/d) for an additional 48 weeks in open label. The patients were followed up at the 12th, 24th, 36th, and 48th weeks (20th, 32nd, 44th, and 56th weeks in the placebo-switching-to-thalidomide group) and the therapeutic efficacy and adverse events were recorded. To minimize the sedative effect of thalidomide, thalidomide was recommended to be taken before sleep. In addition, immunosuppressive agents were prohibited during the whole process of the clinical trial. Patients taking glucocorticoids before recruitment were asked to make a gradual reduction in glucocorticoids with a weekly decline of 5 mg until drug discontinuation and the additional use of glucocorticoids was prohibited.

Withdrawal criteria: (i) loss to follow-up or request for withdrawal by patients themselves; (ii) unintended pregnancy; (iii) intolerable adverse events; (iv) unsuitable for participating in the current clinical trial as determined by the investigators.

### Assessment of index

The patients were visited at the end of the 0, 4th, and 8th weeks during the double-blind phase and 12th, 24th, 36th, and 48th weeks (20th, 32nd, 44th, and 56th weeks in the placebo-switching-to-thalidomide group) during the open-label phase for examining clinical symptoms and signs (e.g. general physical conditions, abdominal pain, diarrhea, etc.), laboratory test results (e.g. blood test, C-reactive protein [CRP], erythrocyte sedimentation rate [ESR], albumin, liver function, etc.), body mass index (BMI), CDAI scores, and inflammatory bowel disease questionnaire (IBDQ) scores. In detail, endoscopy was performed at baseline and 12th and 48th weeks (20th and 56th weeks in the placebo-switching-to-thalidomide group) for recording the simple endoscopic score for Crohn’s disease (SES-CD).

#### Primary outcome

The primary outcome was considered as the clinical remission rate calculated based on the CDAI score at the eighth week following thalidomide (100 mg/d) or placebo treatment. Clinical remission was defined as CDAI falling to <150 points.

#### Secondary outcomes

Secondary outcomes included CDAI scores, the response rate, CRP, and ESR at the 4th and 8th weeks, SES-CD scores, the presence of healed mucosa at the 12th week (20th week in the placebo-switching-to-thalidomide group), and the incidence of adverse events.

Disease response was defined as a minimal decline in CDAI of 100 points or CDAI <150 points. SES-CD scores calculated by colonoscopy of 0–2 indicated healed mucosa.

Any adverse event at each visit was comprehensively assessed, including medical history, vital signs, physical examination, laboratory tests, etc. Because peripheral neuropathy was an adverse event associated with thalidomide use, a complete examination was performed using a standard assessment sheet, particularly for signs and symptoms associated with peripheral neuritis. Moreover, electromyography was performed at Week 8 for peripheral neuropath. In particular, sensory and motor conduction of the median nerve, ulnar nerve, peroneal nerve, and sural nerve were assessed by electromyography. Thalidomide was discontinued if refractory CD patients had both clinical symptoms and electromyographic changes at the same time; otherwise, they were independently monitored by neurologists. Severe adverse events were defined as events during the study that required hospitalization or extended hospital stay, caused disability or impaired ability to work, were life-threatening, death, or congenital malformations.

#### Other index

Human chorionic gonadotropin blood test was performed before recruitment and at every visit for all females of childbearing age. Thalidomide should be discontinued immediately once pregnancy is determined and the induction of labor should be assessed by an obstetrician or a gynecologist.

### Sample size

According to previous studies on thalidomide treatment for CD, we assumed that the clinical remission rate at the eight week was 60% in the thalidomide group and 20% in the placebo group. Then, a total of 46 refractory CD patients were needed in the present study based on the criteria of α = 0.05, β = 0.2 by using Fisher's precision probability test. Following a withdrawal rate of 10%, a total of 50 patients were recruited.

### Random and blind method

A randomization list with blocks of two was computer-generated by an independent team of researchers. Thalidomide and placebo were independently produced by a pharmaceutical factory (Changzhou Pharmaceutical Factory, China) and sequentially labeled with the same appearance and dose (25 mg/tablet), which could not be distinguished from the drug container. All investigators and patients were blinded for the research and outcomes during the first 8 weeks.

### Informed consent and ethics approval

All recruited patients were informed about the risk of teratogenicity, and contraception was required among female and male patients of reproductive potential until 6 months after the trial had ended. All patients signed a written informed consent before the trial.

This clinical trial was approved by the Ethics Committee of the Sixth Affiliated Hospital, Sun Yat-sen University (E2016023) and registered in the National Institutes of Health (NCT02998827).

### Statistical analysis

SPSS 26.0 was used for statistical analyses. An intention-to-treat analysis was performed for every subject. Measurement data were expressed as mean ± standard deviation and normally distributed paired data were compared using the paired *t*-test, or otherwise unpaired two-samples Wilcoxon test or Wilcoxon matched-pairs signed rank test was performed. Enumeration data were expressed as absolute value and percentage. Paired enumeration data were compared using the Fisher's exact test or Yates' chi-square test; otherwise, the McNemar exact test was performed. The correlation between baseline characteristics and clinical remission was assessed by introducing the multivariate Logistic regression model. Two-tailed *P *<* *0.05 was considered as statistically significant.

## Results

### Baseline characteristics

**Figure 1.**
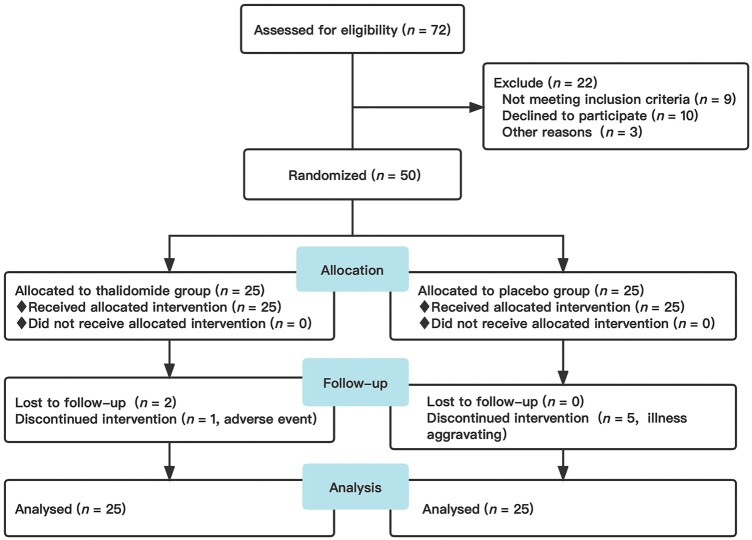
The follow-up diagram of the study

A total of 50 refractory active CD patients were randomly assigned to the thalidomide group (*n *=* *25) and the placebo group (*n *=* *25). In the thalidomide group, 22 (88%) patients completed the 8-week intervention (1 withdrew because of adverse events and 2 were lost to follow-up due to insufficient compliance). In the placebo group, five patients dropped out of the trial because of disease aggravation. The allocation of patients is depicted in Figure 1. There were no significant differences in age, sex, disease course, involved lesions, CD behaviors, surgical or medication history, CRP, ESR, BMI, IBDQ, and SES-CD scores in the thalidomide group and the placebo group (all *P *>* *0.05, Table 1).

**Table 1.** Baseline demographic and clinical characteristics of the patients with refractory Crohn's disease

Characteristic	Thalidomide (*n *=* *25)	Placebo (*n *=* *25)	*P*-value
Age, mean ± SD, years	31.2 ± 11.7	33.7 ± 11.4	0.452
Female, *n* (%)	10 (40.0)	13 (52.0)	0.571
Disease duration, median (IQR), years	4.0 (2.6–5.0)	4.0 (1.1–6.0)	0.414
Involved area, *n* (%)			0.416
Only ileum	3 (12.0)	4 (16.0)	
Only colon	2 (8.0)	3 (12.0)	
Ileum and colon	20 (80.0)	18 (72.0)	
Concomitant upper digestive tract	5 (20.0)	1 (4.0)	
Disease behavior, *n* (%)			0.439
Non-stricturing/non-penetrating	16 (64.0)	20 (80.0)	
Stricturing	8 (32.0)	4 (16.0)	
Concomitant fistula	1 (4.0)	1 (4.0)	
Concomitant perianal disease	10 (40.0)	16 (64.0)	
Previous therapy, *n* (%)			0.811
Steroids	12 (48.0)	18 (72.0)	
Mercaptopurine/azathioprine	19 (76.0)	22 (88.0)	
Infliximab	10 (40.0)	9 (36.0)	
5-Aminosalicylates	10 (40.0)	15 (6.0)	
Enteral nutrition	6 (24.0)	4 (16.0)	
Abdominal surgery	4 (16.0)	3 (12.0)	
Crohn's disease activity index, mean ± SD	234.9 ± 103.7	219.1 ± 57.1	0.506
Inflammatory bowel disease questionnaire score, mean ± SD	157.6 ± 28.5	155.7 ± 22.5	0.852
C-reactive protein, mean ± SD, mg/L	22.1 ± 17.6	21.4 ± 26.1	0.924
Erythrocyte sedimentation rate, mean ± SD, mm/h	41.0 ± 32.0	35.3 ± 22.3	0.463
Body mass index, mean ± SD	18.8 ± 2.6	18.7 ± 2.8	0.916
Simple endoscopic score for Crohn’s disease, mean ± SD	13.7 ± 7.6	16.1 ± 8.8	0.305

SD, standard deviation; IQR, interquartile range.

### Double-blind phase

At the end of the eighth week, the clinical remission rate in the thalidomide group was significantly higher than that in the placebo group (68.0% [17/25] vs 16.0% [4/25]; RR [relative risk], 4.2; 95% confidence interval [CI], 1.8–10.9, *P *<* *0.001). The mean CDAI score, clinical response rate, and decline of CRP were significantly higher in the thalidomide group at the eighth week compared with those in the placebo group (*P *<* *0.05). Although the above indexes were superior in the thalidomide group at the fourth week to those in the placebo group, no significant differences were obtained (*P *<* *0.05) (Table 2).

**Table 2.** The efficacy data in thalidomide and placebo groups

Characteristic	Thalidomide (*n *=* *25)	Placebo (*n *=* *25)	RR (95% CI)	*P*
Outcome at Week 8				
Remission, *n* (%)	17 (68)	4 (16)	4.2 (1.8–10.9)	<0.001
Response, *n* (%)	19 (76)	4 (16)	3.8 (1.8–8.8)	<0.001
CDAI score, mean ± SD	115.6 ± 91.2	196.4 ± 85.5		0.004
Change in ESR, mean ± SD, mm/h	−3.5 ± 22.4	4.4 ± 12.5		0.217
Change in CRP, mean ± SD, mg/L	−9.4 ± 20.6	5.1 ± 23.6		0.015
Outcome at Week 4				
Remission, *n* (%)	10 (40)	6 (24)	1.7 (0.7–3.9)	0.363
Response, *n* (%)	10 (40)	6 (24)	1.7 (0.7–3.9)	0.363
CDAI score, mean ± SD	181.5 ± 81.95	187.2 ± 90.4		0.824
Change in ESR, mean ± SD, mm/h	−1.5 ± 24.1	1.6 ± 18.2		0.636
Change in CRP, mean ± SD, mg/L	−1.1 ± 31.3	2.2 ± 18.0		0.880

RR, relative risk; CI, confidence interval; CDAI, Crohn's disease activity index; SD, standard deviation; ESR, erythrocyte sedimentation rate; CRP, C-reactive protein.

The incidence of adverse events during the double-blind phase was higher in the thalidomide group than that in the placebo group (52.0% [13/25] vs 12.0% [3/25]; RR, 4.3; 95% CI, 1.57–13.12; *P *=* *0.005), while the incidence of serious adverse events showed no significant difference between the two groups (*P *=* *0.992) (Table 3).

**Table 3.** The adverse events in thalidomide and placebo groups

Event	Thalidomide (*n *=* *25)	Placebo (*n *=* *25)	*P*
Adverse events, *n* (%)	13 (52.0)	3 (12.0)	0.005
Severe adverse events leading to discontinuation of treatment, *n* (%)			
Dermatitis	1 (4.0)	0	0.992
Severe adverse events not leading to discontinuation of treatment, *n* (%)	0	0	
Minor adverse events, *n* (%)	12 (48.0)	3 (12.0)	0.012

### Open-label phase

Sixteen CD patients in the placebo group who did not achieve clinical remission at the eighth week were additionally treated with thalidomide for another 8 weeks in the open-label trial and eight (50.0%) achieved clinical remission in the end.

Response CD patients in the thalidomide group and those in the placebo-switching-to-thalidomide group were followed until the 48th week. The overall clinical remission rate of patients (25 in the thalidomide group and 16 in the placebo-switching-to-thalidomide group) at the 12th week was 60.9% (25/41). A total of seven patients achieved mucosal healing (five in the thalidomide group and two in the placebo-switching-to-thalidomide group). The SES-CD score after a 12-week treatment of thalidomide significantly decreased compared with that at baseline (10.57 ± 8.93 vs 14.68 ± 8.20, *P *=* *0.038). Finally, 80.5% (33/41) of patients medicated with thalidomide in the long-term follow-up achieved clinical remission, with a mean duration of 5.9 ± 4.7 weeks. The continuous treatment rate of thalidomide of patients in the thalidomide group and the placebo-switching-to-thalidomide group at the 48th week was 48.0% (12/25) and 43.8% (7/16), respectively; mucosal healing was maintained in 8.0% of patients (2/25) in the thalidomide group and 6.3% (1/16) in the placebo-switching-to-thalidomide group. There were 6, 10, and 6 CD patients who dropped out due to loss to follow-up, disease aggravation, and adverse events, respectively.

At the end of follow-up, 56.0% (14/25) of CD patients in the thalidomide group developed adverse events and 62.5% (10/16) in the placebo-switching-to-thalidomide group developed adverse events. A total of six patients discontinued medication due to adverse events, including four with peripheral neuropathy, one with rash, and one with dizziness. Drowsiness (11 cases) was the most common adverse event, followed by rash (9 cases), peripheral nerve symptoms (7 cases), and constipation (5 cases), and others included swelling of lower limbs, dizziness, and menstrual disturbance. Among the seven patients with peripheral nerve symptoms, four showed positive electromyography results and were partially or completely alleviated after withdrawal.

## Discussion

Biological agents have improved the clinical outcomes of CD patients losing response to steroids and immunosuppressants. However, some may not respond to biological agents or lose response. In addition, it remains a challenge to treat refractory CD patients developing secondary loss of response [18]. In China and some other developing countries, the use of biological agents is limited by their expensiveness. It has taken on a fresh urgency to explore effective and cheaper drugs for CD. A previous randomized–controlled trial (RCT) has verified that thalidomide can induce clinical remission in children and adolescents with refractory CD [13]. Only open-label trials and retrospective studies have reported the application of thalidomide for refractory CD [14–17]. RCTs on the use of thalidomide in the treatment of refractory CD in adults have not been reported yet.

This study was a double-center, randomized, double-blind, placebo-controlled study. Only thalidomide or placebo was used in the induction and maintenance therapy, which accurately reflected the efficacy of thalidomide on CD. At the end of the double-blind phase, the clinical remission rate of adult refractory CD patients in the thalidomide group was significantly higher than that in the placebo group (68% [17/25] vs 16% [4/25], RR, 4.2; 95% CI, 1.8–10.9; *P *<* *0.001). At 12 weeks, the overall clinical remission rate was 60.9% (25/41). A total of seven refractory CD patients achieved mucosal healing. Concerning the comparable clinical remission rate with current biological agents, thalidomide can be an option for refractory CD patients. Consistently with previous findings, the use of infliximab in refractory CD patients did not influence the time to achieve clinical remission [10, 19]. Although infliximab, adalimumab, and thalidomide all exert their pharmacological functions through suppressing TNF-α, the specific mechanisms may vary a lot [20]. Thalidomide is also found to inhibit the activity of NF-κB and regeneration of small intestinal microvascular endothelial cells, as well as downregulate mucosal vascular addressin cell adhesion molecule-1, which are highly expressed in CD patients [21, 22]. Therefore, induction therapy of thalidomide is an option for refractory CD patients with failed biological agents or those who cannot be medicated with biological agents.

Thalidomide has been removed from the market by the FDA for its teratogenicity. It also causes peripheral neuropathy [23, 24]. In our study, the incidence of adverse events during the double-blind phase was higher in the thalidomide group than that in the placebo group (52.0% [13/25] vs 12.0% [3/25]; RR, 4.3; 95% CI, 1.57–13.12; *P *=* *0.005), while the incidence of serious adverse events showed no significant difference (*P *=* *0.992). At the end of follow-up, 56.0% (14/25) of CD patients in the thalidomide group had developed adverse events and 62.5% (10/16) in the placebo-switching-to-thalidomide group developed adverse events, among whom four had peripheral neuropathy. The incidence of adverse events in the present study was lower than that in other trials [24]. Peripheral neuropathy is commonly observed in multiple myeloma patients treated with high-dose thalidomide combined with chemotherapy. Immunosuppressant-associated adverse events are also observed in the treatment of CD using thalidomide. Other adverse events caused by thalidomide in the treatment of CD included somnolence, dermatitis, and constipation. Although their incidences remained high, they were tolerable without affecting the quality of life. Other adverse events, such as bradycardia, were related to thalidomide treatment. Electromyography should be monitored in patients receiving thalidomide treatment [24]. In our study, two female patients developed symptoms of menstrual disorders. A report has demonstrated amenorrhoea and hypergonadotropic hypogonadism in women taking thalidomide for various diseases [25]. A relevant study conducted in our center has shown that thalidomide treatment diminishes the ovarian reserve in female CD women of reproductive age, which can be reversed several months after drug withdrawal [26]. Amenorrhea was not observed in our study, which may be attributed to the short observation period and the small sample size. We did not identify the risk of thrombosis caused by thalidomide. Nevertheless, thalidomide combined with chemotherapy may increase the risk of thrombosis in the treatment of adult multiple myeloma [27]. A close monitoring of thrombosis, as a result, is required in thalidomide treatment of CD. Whether refractory CD patients during the remission period can reduce the dose of thalidomide to reduce the incidence of adverse reactions should be further investigated.

Thalidomide-induced phocomelia used to be a severe event. In fact, the teratogenic risk of thalidomide can be prevented by contraception. Other drugs for CD also come with adverse events. For example, steroid drugs can disrupt growth and development, and lead to osteoporosis. Immunosuppressants and novel biological agents may increase the risks of infection, cancer, bone marrow suppression, and liver failure [28]. Rational monitoring is expected to control the potential adverse events. In a large-scale thalidomide clinical trial involving 124,000 participants, no major adverse event appeared during the 6-year observation period [29]. Thalidomide has been proven to be safe in the treatment of multiple myeloma, leprosy, and rheumatic diseases [30]. Our study revealed that most of the adverse events of thalidomide could be monitored and controlled, and the incidence of severe adverse events was relatively low. Taken together, the adverse events of thalidomide are tolerable under close monitoring.

CD treatment is expensive. Some biological agents and small-molecule-targeted drugs do improve the prognosis of CD, but their daily cost may be 22 times that of conventional chemical drugs. As a result, their application as a maintenance therapy is significantly restricted. Thalidomide is cheap, tolerable, and effective in inducing clinical remission of refractory CD, turning it into an ideal treatment option in economically underdeveloped areas. But its dose and duration should be carefully controlled. Notably, pregnancy and side effects should be monitored and neurotrophic drugs are recommended to reduce its toxicity to nerves if necessary.

The study has some limitations. First, although it was a prospective randomized–controlled study, the overall sample size was relatively small. Second, the observation period was only 1 year. Whether continued use of thalidomide might lead to more peripheral neuritis needs more observation.

## Conclusions

Thalidomide is effective at inducing and maintaining clinical remission and mucosal healing in refractory CD adults. Adverse events of varying degrees may occur, but most of them can be well controlled. Owing to its low medical cost and high therapeutic efficacy, 100 mg/d thalidomide is recommended in the treatment of refractory CD, especially in economically underdeveloped areas.

## Authors’ Contributions

X.P., Q.C., and M.Zhi were involved in study conception and design; X.P., Z.W.L., and M.Zhang performed literature search and data collection; X.P., J.Y.Y., and J.Z.Z. performed data analysis and interpretation; X.P., M.Zhang, P.J.H., Q.C., and M.Zhi were involved in drafting of the manuscript; X.P., Z.W.L., M.Zhang, J.Y.Y., J.Z.Z., P.J.H, Q.C., and M.Zhi were involved in critical revision of the manuscript for important intellectual content. All authors have approved the final version of the manuscript.

## Funding

This study was supported by Academician Jie-shou Li Intestinal Barrier Special Research Fund [grant number LJS-201908C].

## References

[1] El-Matary W , Moroz SP, Bernstein CN. Inflammatory bowel disease in children of Manitoba: 30 years' experience of a tertiary center. J Pediatr Gastroenterol Nutr 2014;59:763–6. 25111222 10.1097/MPG.0000000000000525

[2] Rutgeerts P , Sandborn WJ, Feagan BG et al Infliximab for induction and maintenance therapy for ulcerative colitis. N Engl J Med 2005;353:2462–76. 16339095 10.1056/NEJMoa050516

[3] Ye XQ , Cai J, Yu Q et al Nomogram to predict primary non-response to infliximab in patients with Crohn's disease: a multicenter study. Gastroenterol Rep (Oxf) 2021;9:329–38. 34567565 10.1093/gastro/goaa069 PMC8460115

[4] Gisbert JP , Panes J. Loss of response and requirement of infliximab dose intensification in Crohn's disease: a review. Am J Gastroenterol 2009;104:760–7. 19174781 10.1038/ajg.2008.88

[5] Billioud V , Sandborn WJ, Peyrin-Biroulet L. Loss of response and need for adalimumab dose intensification in Crohn's disease: a systematic review. Am J Gastroenterol 2011;106:674–84. 21407178 10.1038/ajg.2011.60

[6] Feuerstein JD , Nguyen GC, Kupfer SS et al; American Gastroenterological Association Institute Clinical Guidelines Committee. American Gastroenterological Association Institute guideline on therapeutic drug monitoring in inflammatory bowel disease. Gastroenterology 2017;153:827–34. 28780013 10.1053/j.gastro.2017.07.032

[7] Regan BP , Bousvaros A. Pediatric ulcerative colitis: a practical guide to management. Paediatr Drugs 2014;16:189–98. 24723200 10.1007/s40272-014-0070-8

[8] Van Assche G , Lewis JD, Lichtenstein GR et al The London position statement of the World Congress of Gastroenterology on Biological Therapy for IBD with the European Crohn's and Colitis Organisation: safety. Am J Gastroenterol 2011;106:1594–602; quiz 1593, 1603. 21844919 10.1038/ajg.2011.211

[9] Sheskin J. Thalidomide in the treatment of lepra reactions. Clin Pharmacol Ther 1965;6:303–6. 14296027 10.1002/cpt196563303

[10] Bauditz J , Wedel S, Lochs H. Thalidomide reduces tumour necrosis factor alpha and interleukin 12 production in patients with chronic active Crohn's disease. Gut 2002;50:196–200. 11788559 10.1136/gut.50.2.196 PMC1773116

[11] Plamondon S , Ng SC, Kamm MA. Thalidomide in luminal and fistulizing Crohn's disease resistant to standard therapies. Aliment Pharmacol Ther 2007;25:557–67. 17305756 10.1111/j.1365-2036.2006.03239.x

[12] Tseng S , Pak G, Washenik K et al Rediscovering thalidomide: a review of its mechanism of action, side effects, and potential uses. J Am Acad Dermatol 1996;35:969–79. 8959957 10.1016/s0190-9622(96)90122-x

[13] Lazzerini M , Martelossi S, Magazzu G et al Effect of thalidomide on clinical remission in children and adolescents with refractory Crohn disease: a randomized clinical trial. JAMA 2013;310:2164–73. 24281461 10.1001/jama.2013.280777

[14] Vasiliauskas EA , Kam LY, Abreu-Martin MT et al An open-label pilot study of low-dose thalidomide in chronically active, steroid-dependent Crohn's disease. Gastroenterology 1999;117:1278–87. 10579968 10.1016/s0016-5085(99)70277-5

[15] Ehrenpreis ED , Kane SV, Cohen LB et al Thalidomide therapy for patients with refractory Crohn's disease: an open-label trial. Gastroenterology 1999;117:1271–7. 10579967 10.1016/s0016-5085(99)70276-3

[16] Gerich ME , Yoon JL, Targan SR et al Long-term outcomes of thalidomide in refractory Crohn's disease. Aliment Pharmacol Ther 2015;41:429–37. 25511905 10.1111/apt.13057

[17] Chen J-R , Mai L, Sun J-C et al Efficacy and safety of low-dose thalidomide combined with mesalazine in the treatment of refractory ulcerative colitis in adults. Gastroenterol Rep (Oxf) 2022;10:goac032. 35975242 10.1093/gastro/goac032 PMC9373931

[18] Ng SC , Chan FK, Sung JJ. Review article: the role of non-biological drugs in refractory inflammatory bowel disease. Aliment Pharmacol Ther 2011;33:417–27. 21138457 10.1111/j.1365-2036.2010.04541.x

[19] Sabate JM , Villarejo J, Lemann M et al An open-label study of thalidomide for maintenance therapy in responders to infliximab in chronically active and fistulizing refractory Crohn's disease. Aliment Pharmacol Ther 2002;16:1117–24. 12030953 10.1046/j.1365-2036.2002.01273.x

[20] Felipez LM , Gokhale R, Tierney MP et al Thalidomide use and outcomes in pediatric patients with Crohn disease refractory to infliximab and adalimumab. J Pediatr Gastroenterol Nutr 2012;54:28–33. 21681114 10.1097/MPG.0b013e318228349e

[21] Franks ME , Macpherson GR, Figg WD. Thalidomide. Lancet 2004;363:1802–11. 15172781 10.1016/S0140-6736(04)16308-3

[22] Laffitte E , Revuz J. Thalidomide: an old drug with new clinical applications. Expert Opin Drug Saf 2004;3:47–56. 14680461 10.1517/14740338.3.1.47

[23] Ginsburg PM , Dassopoulos T, Ehrenpreis ED. Thalidomide treatment for refractory Crohn's disease: a review of the history, pharmacological mechanisms and clinical literature. Ann Med 2001;33:516–25. 11730158 10.3109/07853890108995961

[24] Ghobrial IM , Rajkumar SV. Management of thalidomide toxicity. J Support Oncol 2003;1:194–205. 15334875 PMC3134146

[25] Lazzerini M , Bramuzzo M, Martelossi S et al Amenorrhea in women treated with thalidomide: report of two cases and literature review. Inflamm Bowel Dis 2013;19:E10–11. 22161965 10.1002/ibd.22845

[26] Peng X , Zhi M, Wei M et al Thalidomide results in diminished ovarian reserve in reproductive age female IBD patients. Medicine (Baltimore) 2017;96:e6540. 28538364 10.1097/MD.0000000000006540 PMC5457844

[27] Fabi SG , Hill C, Witherspoon JN et al Frequency of thromboembolic events associated with thalidomide in the non-cancer setting: a case report and review of the literature. J Drugs Dermatol 2009;8:765–9. 19663116

[28] de Silva S , Devlin S, Panaccione R. Optimizing the safety of biologic therapy for IBD. Nat Rev Gastroenterol Hepatol 2010;7:93–101. 20134491 10.1038/nrgastro.2009.221

[29] Uhl K , Cox E, Rogan R et al Thalidomide use in the US : experience with pregnancy testing in the S.T.E.P.S. programme. Drug Saf 2006;29:321–9. 16569081 10.2165/00002018-200629040-00003

[30] Morgan GJ , Davies FE. Role of thalidomide in the treatment of patients with multiple myeloma. Crit Rev Oncol Hematol 2013;88 Suppl 1:S14–22. 23827438 10.1016/j.critrevonc.2013.05.012

